# Sociality, ecology and developmental constraints predict variation in brain size across birds

**DOI:** 10.1111/jeb.14117

**Published:** 2022-11-10

**Authors:** Jasmine L. Hardie, Christopher R. Cooney

**Affiliations:** ^1^ Ecology and Evolutionary Biology, School of Biosciences University of Sheffield Sheffield UK

**Keywords:** birds, brain, brain size, developmental mode, ecology, sociality

## Abstract

Conflicting theories have been proposed to explain variation in relative brain size across the animal kingdom. Ecological theories argue that the cognitive demands of seasonal or unpredictable environments have selected for increases in relative brain size, whereas the ‘social brain hypothesis’ argues that social complexity is the primary driver of brain size evolution. Here, we use a comparative approach to test the relative importance of ecology (diet, foraging niche and migration), sociality (social bond, cooperative breeding and territoriality) and developmental mode in shaping brain size across 1886 bird species. Across all birds, we find a highly significant effect of developmental mode and foraging niche on brain size, suggesting that developmental constraints and selection for complex motor skills whilst foraging generally imposes important selection on brain size in birds. We also find effects of social bonding and territoriality on brain size, but the direction of these effects do not support the social brain hypothesis. At the same time, we find extensive heterogeneity among major avian clades in the relative importance of different variables, implying that the significance of particular ecological and social factors for driving brain size evolution is often clade‐ and context‐specific. Overall, our results reveal the important and complex ways in which ecological and social selection pressures and developmental constraints shape brain size evolution across birds.

## Graphical abstract

Across all birds we found significant effects of developmental mode, foraging niche, social bonding and territoriality on relative brain size. This suggests that developmental constraints, selection for complex motor skills whilst foraging, and social factors generally imposes important selection on avian brain size. However, we also found extensive heterogeneity among major avian clades in the relative importance of different variables, implying that the significance of particular ecological and social factors for driving brain size evolution is often clade‐ and context‐specific.

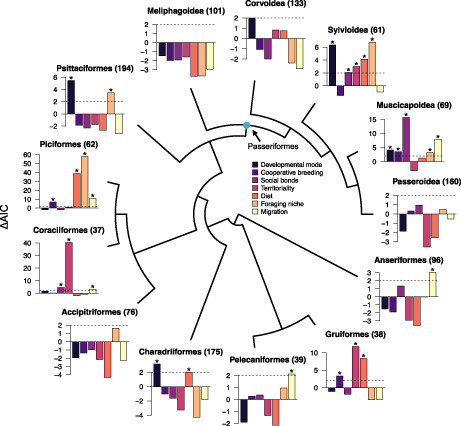

## 1 INTRODUCTION

Understanding how and why animals differ in their cognitive abilities is a central tenet of research in behavioural ecology, but difficulties in defining and measuring cognitive complexity have limited advances in our understanding. Relative (i.e. body mass‐adjusted) brain size is a popular proxy for cognitive ability, and correlates with numerous behaviours associated with complex cognition, including tool use (Lefebvre et al., 2002), play (Iwaniuk et al., 2001), social learning (Reader & Laland, 2002), and innovation (Overington et al., 2009). Implicit within theories of brain size evolution is the assumption that increases in relative brain size confer an increase in cognitive function, and thus that relatively large‐brained species are capable of more complex behaviours (Healy & Rowe, 2007).

Alternative theories have been proposed to explain variation in relative brain size and there are a number of comparative analyses investigating evolutionary drivers of brain size (Healy & Rowe, 2007). Many of these studies focus on either ecological (e.g. ‘cognitive buffer hypothesis’) (Ducatez et al., 2015; Overington et al., 2011; van Woerden et al., 2012; Winkler et al., 2004) or social (e.g. ‘social brain hypothesis’) (Beauchamp & Fernández‐Juricic, 2004; Iwaniuk & Arnold, 2004; Shultz & Dunbar, 2007; West, 2014) theories of brain size evolution. The cognitive buffer hypothesis predicts that species with relatively large brains should exhibit greater behavioural plasticity and thus be better at overcoming challenges relating to resource acquisition and/or unpredictable environments (Sol, 2009). The social brain hypothesis argues that selection for behavioural plasticity increases with social complexity, and that consequently social environment is the primary driver of brain size (Dunbar, 1998). Both the cognitive buffer hypothesis and the social brain hypothesis posit that increases in brain size have resulted from selection for increased behavioural flexibility, but differ in the factors (i.e. ecological vs. social) that they emphasize as driving the evolution of brain size. However, despite a growing body of comparative research, we still lack a clear understanding of the relative importance of social and ecological drivers of brain size.

One reason for this is that many studies fail to integrate both social and ecological variables into their analysis (Healy & Rowe, 2007). A game theoretical model found that resource distribution affects both the degree of foraging generalism and the likelihood of social foraging (Overington et al., 2008). This suggests that the cognitive requirements of sociality and ecology may not be independent from one another and emphasizes the importance of incorporating both sociality and ecology into studies of brain size evolution. In terms of empirical studies, DeCasien et al. (2017) investigated social and ecological predictors of primate brain size and found that brain size was predicted by diet but not sociality. The authors speculate that frugivory selects for greater cognitive complexity because frugivores often use extractive foraging and must retain and process spatial information on food distribution. However, the relative importance of dietary versus foraging mode differences in shaping primate brain size was not directly addressed. In contrast, Shultz and Dunbar (2006) found that both sociality and ecology drive evolution in ungulate brain size, suggesting that the relative importance of social/ecological drivers of brain size may vary between taxa.

Taxonomic biases in studies of the social brain hypothesis and anthropocentric definitions of sociality may also limit our understanding of social drivers of brain size evolution across diverse taxa (Barrett et al., 2007). The social brain hypothesis was originally developed in primates, after it was suggested that complex social dynamics within multi‐male, multi‐female may have driven the evolution of relatively large brains within this taxon (Dunbar, 1998). Consequently, much of the research on social drivers of cognitive evolution has focused on primates (e.g. Barton, 1996; DeCasien et al., 2017; Sandel et al., 2016; Shultz & Dunbar, 2007). However, the theory underlying the social brain hypothesis—that is, social complexity drives brain size evolution—can be applied to other taxa that have diverse social systems (Barrett et al., 2007). Birds are a useful taxon for testing the generality of the social brain hypothesis while simultaneously examining ecological effects on brain size because although their social systems differ from those of primates in important ways (Dunbar & Shultz, 2007), avian social systems nonetheless range from primarily solitary, with uniparental care, to highly social with year‐round associations and cooperative care of young (Tobias et al., 2016). A previous study found no association between group size and brain size in birds (Beauchamp & Fernández‐Juricic, 2004), but group size is a relatively crude measure of sociality in birds, as many species aggregate in large flocks, which may not reflect an increase in social complexity. Downing et al. (2020) found that differences in the way groups formed and the relatedness of group members predict the level of social complexity and Shultz and Dunbar (2010) found that social bond was associated with relative brain size in birds. Therefore, group size may not be an effective proxy for social complexity in birds, and more nuanced, taxonomically relevant measures of social complexity are required to investigate social drivers of brain size evolution.

The aim of this study is to investigate the relative importance of social and ecological drivers of brain size evolution across bird species. To do this, we focus on three key measures of ecology (foraging niche, diet and migration) and three key measures of sociality (social bond, cooperative breeding and territoriality). Previous research on ecological correlates of avian brain size evolution finds that species with larger brains relative to their body size utilize more innovative, generalist foraging strategies (Overington et al., 2009). Producing and maintaining large brains is energetically costly (Mink et al., 1981). The ‘expensive brain hypothesis’ suggests the costs of expensive brain tissue can be met either by increasing energy intake or through trade‐offs in energy investment (Isler & van Schaik, 2009). Species with a higher calorie diet or that forage on more reliable or abundant food types are, therefore, more likely to be able to satisfy the energy requirements of relatively large brains. On the other hand, species that undergo long migrations have high energy requirements and are thus less likely to be able to maintain large brains relative to their size (Winkler et al., 2004). We, therefore, hypothesized that variation in both foraging niche and diet would correlate with differences in avian brain size across species, and that migration would be negatively correlated with brain size, with more sedentary species exhibiting relatively larger brains.

On the other hand, the social brain hypothesis predicts that social complexity is the primary driver in the evolution of relatively large brains (Dunbar, 1998). Cooperative breeding systems are widely considered to demonstrate social complexity in birds (e.g. Cornwallis et al., 2010; Downing et al., 2020) and territoriality, where individuals defend foraging or nesting sites, is widespread across the radiation. Bird song plays an important role in territory defence for many bird species (Naguib & Riebel, 2014), and territorial songbirds have an increased auditory song memory capacity (Ikebuchi & Okanoya, 2000). In order to avoid costly interactions with conspecifics when foraging or courting, many territorial species may also benefit from an increased spatial memory capacity (Potts & Lewis, 2016) and it has been shown that a better spatial memory increases the likelihood of territory ownership in a territorial hummingbird (Araya‐Salas et al., 2018). Therefore, we hypothesized that the strength of social bonding, presence of cooperative breeding, and degree of territoriality would all exhibit positive associations with relative brain size.

Finally, previous studies have identified variation in developmental mode as an important predictor of relative brain size across birds, with fast‐developing precocial species typically possessing smaller brains relative to slower‐developing altricial species in which offspring have longer periods of embryonic development (Bennett & Harvey, 1985; García‐Peña et al., 2013; Iwaniuk & Nelson, 2003; Shultz & Dunbar, 2010; Starck & Ricklefs, 1998). Therefore, we also incorporated information on species' developmental mode into our analyses to control for possible differences in developmental constraints across species that may covary with ecological or social factors.

## 2 METHODS

### 2.1 Trait data

As outlined above, we identified a number of social and ecological predictor variables that could drive interspecific variation in avian brain size. Data sources are described in detail below, and species with incomplete data were excluded from the analysis.

#### 2.1.1 Brain size

Brain volume data were obtained from Ksepka et al. (2020).This dataset combines lead‐shot endocast data (Sayol et al., 2018) with CT‐rendered endocast measurements of brain volume. Endocasts measure the volume of the endocranial cavity, and are a reliable proxy for brain size in birds (Iwaniuk & Nelson, 2002). Both lead‐shot and CT‐rendered endocasts have been widely used as proxies for brain size (Balanoff et al., 2016; Iwaniuk & Nelson, 2002) so variation in the endocast methodology used is unlikely to be an important source of error in our results. We initially considered 1980 extant species with brain size data. This number was reduced to a final dataset of 1886 species due to missing data for predictor variables (94 species lacked information on sociality).

#### 2.1.2 Body mass

Body mass is closely correlated with brain size in birds due to strong allometric effects (Sol & Price, 2008). Therefore, to remove the allometric effects of body size on brain size, we calculated relative (i.e. mass‐adjusted) brain size values using established approaches (see below). Body mass data were taken from Ksepka et al. (2020), who collated data from a compendium on avian body mass (Dunning, 2008). Where the sex of the specimens used for brain volume estimates was known, sex‐specific body mass was provided. Otherwise, the average body mass for both sexes was used.

#### 2.1.3 Ecological variables

Information on diet, foraging niche and migration were all taken from Tobias and Pigot (2019). In this dataset, species are assigned to one of 10 dietary categories (aquatic animals, aquatic plants, terrestrial invertebrates, terrestrial vertebrates, terrestrial carrion, nectar, seeds, fruit, other terrestrial plant matter, or omnivore), and to one of nine foraging niches (aerial screen, bark glean, aerial sally, arboreal glean, ground forage, aquatic plunge, aquatic surface, aquatic dive, or generalist [if a species used multiple foraging strategies]). For descriptions of each foraging and dietary category, and information on how species were assigned to categories, please see Tobias and Pigot (2019). While this classification scheme provides higher resolution information on species' ecological niche differences than similar datasets (e.g. EltonTraits [Wilman et al., 2014]), the relatively large number of categories increases the complexity of downstream statistical models without necessarily providing additional insight. Therefore, following previous studies (e.g. Felice et al., 1897) we explored a simpler classification scheme in which related categories were collapsed into broader categories. Specifically, we collapsed the Tobias and Pigot dataset into six broader dietary categories: ‘aquatic animals’, ‘fruit/nectar’, ‘generalist’, ‘invertebrates’, ‘plants/seeds’, ‘vertebrates’. Similarly, for foraging niche we collapsed the dataset into six broader categories: ‘aerial sallying’, ‘aerial screening’, ‘aquatic foraging’, ‘foraging generalist’, ‘gleaning’, ‘ground foraging’. In the main text we focus on analyses based on our simplified ecological categories, with analyses based on the original, more complex classification scheme presented as supplementary results. Migratory behaviour was categorized as either ‘sedentary’, ‘partially migratory’ (some individuals exhibit long‐distance migration or majority of population migrates over short distances), or ‘migratory’ (majority of the population exhibits long‐distance migration).

#### 2.1.4 Social variables

Social bond data were obtained from Tobias et al. (2016). Species were classified as either ‘solitary’ (forming only temporary associations between sexual partners during courtship and with uniparental care), ‘weakly social’ (weak/seasonal sociality between breeding partners with low mate fidelity and a high [>50% per annum] divorce rate across breeding seasons), or ‘highly social’ (either year‐round associations between breeding partners or high mate fidelity and a low [<50% per annum] divorce rate across breeding seasons). Regarding our social bond data, we note that this classification scheme focuses on sociality between breeding partners and does not capture other potentially important axes of social complexity that occur outside of breeding interactions (e.g. flocking, flock fusion/fission propensity). Territoriality and cooperative breeding data were from Tobias and Pigot (2019). Species were classified as either ‘cooperative’ or ‘non‐cooperative’ according to the breeding systems described by Jetz and Rubenstein (2011). Territoriality was scored as either ‘none’ (never territorial or at most defending very small areas around nest sites), ‘weak’ (weak or seasonal territoriality, including species with broadly overlapping home ranges or habitually joining mixed species flocks), or ‘strong’ (territories maintained throughout year). For full details on social variable classification schemes and definitions, see Tobias et al. (2016).

#### 2.1.5 Developmental mode

Quantitative data on variation in developmental mode across species were obtained from a recently published analysis based on measurements for multiple hatchling and post‐hatching traits from thousands of bird species (Ducatez & Field, 2021). We focused on the most integrative index of avian developmental mode provided by the authors (‘Chick PC1’), which provides a unique quantitative estimate of species' position along the avian altricial‐precocial spectrum (altricial species have negative scores, precocial species have positive scores).

### 2.2 Phylogeny

**FIGURE 1:**
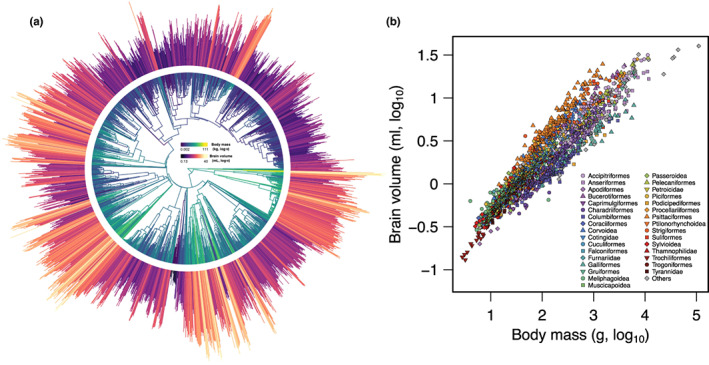
The phylogenetic distribution and allometry of brain volume in birds. (a), Coloured branches and bars at the tips of the phylogeny indicate relative body mass and brain volume, respectively, for 1886 bird species. Internal branch colours corresponding to ancestral body mass estimates are for visualization purposes only. (b), The allometric relationship between (log_10_‐transformed) values of brain volume and body mass, with major taxonomic groups (>10 spp.) indicated.

Our analyses are based on the taxonomy and phylogenies developed by Jetz et al. (2012). To provide a phylogenetic framework for the species included in our analysis (*n* = 1886), we downloaded 100 trees from the posterior distribution of complete trees produced by Jetz et al. (2012) from http://www.birdtree.org. These trees were then pruned to generate a distribution of trees containing only the focal species set. All of our analyses were run over this distribution of 100 trees in order to incorporate phylogenetic uncertainty into our parameter estimates. For plotting purposes (i.e. Figure 1a), we identified a maximum clade credibility (MCC) tree from this posterior distribution of trees using the ‘maxCladeCred’ function in the R package ‘phangorn’ (version 2.5.5) (Schliep, 2011).

### 2.3 Statistical analysis

All statistical analyses were carried out using R version 4.0.2. Brain volume and body mass values were log_10_‐transformed prior to analyses. To test for relationships between our variables, we used phylogenetic generalized least‐squares (PGLS) regression with an optimized lambda error structure in all cases, which controls for phylogenetic non‐independence of species' values by adjusting for the observed level of phylogenetic signal in the residuals of the model (Symonds & Blomberg, 2014). PGLS models were implemented in the R package ‘phylolm’ (Ho & Ané, 2014) using model = ‘lambda’ with all other settings using default values.

To remove the allometric effects of body size on brain size, we followed existing studies (Sayol et al., 2018, 2019) and estimated relative brain size using the residuals from a log–log PGLS regression of brain volume against body mass. Previous analyses have clearly demonstrated evolutionary shifts in the allometric relationship between brain and body size among major avian clades (Ksepka et al., 2020). Therefore, to avoid assuming a single allometric relationship across all birds, which can introduce bias into relative brain size estimates, we fit a series of more complex models in which the intercept and slope of the brain‐to‐body relationship was allowed to vary among taxonomic groups. We focused on groups represented by 10 or more species in our dataset that correspond to non‐passerine orders and major passerine subgroups (Cooney et al., 2017). The best‐supported model was one in which the intercept and slope of the brain‐to‐body size relationship are allowed to vary among groups (Tables S1 and S2). We, therefore, used this model as the basis for estimating species' relative brain size in our analysis. We also explored an alternative approach for controlling for allometric effects on brain size that involved including body mass as an additional covariate in multi‐predictor models of brain volume. Although this alternative approach does not control for variable allometric relationships across clades as our primary approach does, we found that results were similar across both methods. Therefore, in the main text we focus on results based on clade‐specific relative brain size estimates and present body mass covariate results as supplementary results.

To assess the overall importance of sociality, ecology and development for explaining relative brain size evolution across birds as a whole, and to assess the extent to which any such overarching effects are mirrored and/or differ among avian clades, we tested relationships between brain size and our predictor variables at two taxonomic levels: (1) across all birds (i.e. all species in our dataset) and (2) within major avian clades. For our clade analyses, clades (see above) were retained for analysis if they (i) were represented in our dataset by >30 species and (ii) exhibited variation among species in all focal variables, thereby providing the power for us to test our hypotheses. In total, 13 clades (8 non‐passerine, 5 passerine) met these criteria, encompassing 1241 (66%) species in our dataset, ranging in size from 37 to 194 species. Analyses were conducted similarly at both taxonomic levels, except that for clades we re‐estimated relative brain size values using individual PGLS models of brain against body size, rather than relying on the residuals derived from the broader, whole‐tree model.

Throughout our analysis, predictors were deemed important if model support values dropped by >2 units (i.e. ΔAIC > 2) when the predictor was dropped from the model while holding lambda values constant (Cooney et al., 2020). Larger ΔAIC values indicate greater statistical support for the importance of a predictor (Burnham & Anderson, 2002) and here we focused only on the main effects of predictors on brain size (i.e. we did not examine interaction effects). Partial‐*R* ^2^ values associated with predictors were calculated using the ‘R2.lik’ function in the R package ‘rr2’ (Ives, 2019). Finally, phylogenetic signal values (i.e. Pagel's *λ* values [Pagel, 1999]) were calculated by fitting an intercept‐only model to each trait using phylolm and with boot = 100. In all cases, parameter estimates represent mean values across the distribution of 100 posterior trees.

## 3 RESULTS

Figure 1a shows the phylogenetic distribution of brain volume and body mass variation across the 1886 bird species included in our analyses. Both traits exhibit very strong phylogenetic signal, with *λ* values of 0.987 (95% confidence interval: 0.981–0.991) for brain volume, 0.982 (0.976–0.988) for body mass and 0.869 (0.838–0.895) for relative (i.e. mass‐corrected) brain size. As demonstrated by previous studies, there is a strong positive allometric relationship between brain and body size in birds (Figure 1b), but with clear shifts among clades in the relative intercept and slope of the brain–body size relationship (Tables S1 and S2).

**FIGURE 2:**
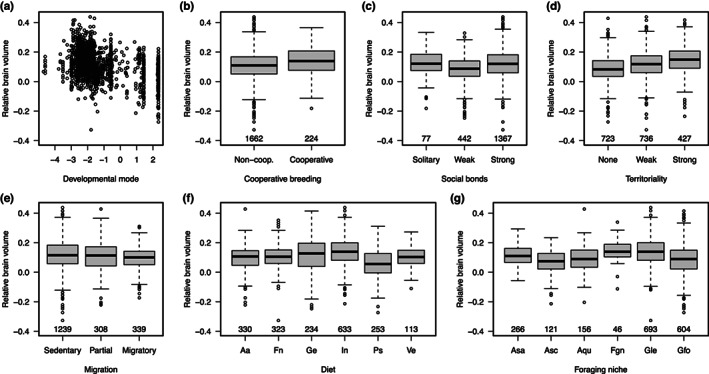
Plots showing the relationship of relative brain volume with developmental, social and ecological variables across bird species. In all cases, box and whisker plots show the median (centre line) and interquartile range (box) of the data, the range of data, which is within 1.5 times the interquartile range of the box (whiskers), and the position of outliers (points) that lie beyond this range. Values under each box give the number of species in each category. In F, letter codes represent ‘aquatic animals’ (Aa), ‘fruit/nectar’ (Fn), ‘generalist’ (Ge), ‘invertebrates’ (In), ‘plants/seeds’ (Ps), ‘vertebrates’ (Ve). In G, letter codes represent ‘aerial sallying’ (Asa), ‘aerial screening’ (Asc), ‘aquatic’ (Aqu), ‘foraging generalist’ (Fgn), ‘gleaning’ (Gle), ‘ground foraging’ (Gfo).

After accounting for these allometric effects, across all birds we found significant relationships between relative brain size and several of our predictor variables (Table 1; see Figure 2 for the number of species across ecological and social categories). First, we detected a strong relationship between relative brain size and variation in developmental mode across species (ΔAIC = 17.9): species with highly precocial development have significantly smaller brains than species with altricial development (Figure 2a, Table 1).

**TABLE 1:** Phylogenetic generalized least‐squares model results for the effect of developmental, ecological and social predictor variables on relative brain volume across avian species (*n* = 1886).

Term	Level	Estimate (SE)	*p*	ΔAIC	Δ*R* ^2^
(Intercept)	–	0.021 (0.038)	0.595		
Developmental mode	–	−0.020 (0.005)	<0.001	**17.893**	**0.010**
Cooperative breeding	Cooperative	−0.010 (0.005)	0.069	1.429	0.002
Social bond	Weakly social	−0.026 (0.011)	0.019	**13.041**	**0.009**
	Highly social	−0.009 (0.011)	0.401		
Territoriality	Weak	0.002 (0.005)	0.655	**5.960**	**0.005**
	Strong	0.017 (0.007)	0.013		
Diet	Fruit/nectar	−0.012 (0.009)	0.189	−4.586	0.003
	Generalist	−0.008 (0.008)	0.350		
	Invertebrates	−0.003 (0.008)	0.714		
	Plants/seeds	−0.014 (0.008)	0.101		
	Vertebrates	0.010 (0.013)	0.455		
Foraging niche	Aerial screening	−0.017 (0.010)	0.095	**15.314**	**0.013**
	Aquatic foraging	0.026 (0.013)	0.053		
	Generalist	0.011 (0.011)	0.315		
	Gleaning	0.030 (0.008)	0.001		
	Ground foraging	0.016 (0.009)	0.060		
Migration	Partially migratory	−0.002 (0.005)	0.601	1.012	0.003
	Migratory	−0.011 (0.005)	0.031		

*Note*: Parameter estimates represent mean values from models run over 100 posterior phylogenetic trees. For categorical variables, the reference categories are ‘Non‐cooperative’ (Breeding system), ‘Solitary’ (Social bond), ‘None’ (Territoriality), ‘Aquatic animals’ (Diet), ‘Aerial sallying’ (Foraging niche) and ‘Sedentary’ (Migration). SE, standard error. Variables exhibiting values of ΔAIC ≥ 2 are highlighted in bold.

In terms of our social variables, we found that both social bond type (ΔAIC = 13.0) and degree of territoriality (ΔAIC = 6.0) explained a significant proportion of variation in relative brain size across birds. However, contrary to our hypothesis, weakly social species had significantly smaller brains than solitary species, with less pronounced differences between brain sizes of solitary and highly social species (Figure 2c, Table 1). For territoriality, in line with our prediction we found that highly territorial species had significantly larger brains than non‐territorial species (Figure 2d, Table 1). In contrast to the effects of social bonding and territoriality, the relationship between cooperative breeding and brain size was non‐significant (ΔAIC = 1.4).

Regarding our ecological variables, only variation in foraging niche (ΔAIC = 15.3) explained a significant proportion of variation in relative brain size across all species (Figure 2g, Table 1). Parameter estimates revealed that, after controlling for the effects of phylogeny and other predictor variables, gleaning, generalist and aquatic foraging modes were associated with relatively larger brains, whilst species foraging via aerial sallying and aerial screening tended to have smaller brains (Table 1). In contrast, across all birds there was no significant effect of migration on relative brain size (ΔAIC = 1.0; Figure 2g). Similarly, after controlling for other factors we found no evidence that dietary niche differences explained a significant proportion of variation in brain size across species (ΔAIC = −4.6) despite variation in relative brain size across dietary categories (Figure 2e), as relative model fit improved when this predictor was excluded from the analysis (Table 1). We note that analyses including body mass as a covariate and/or using finer ecological categorizations produced similar results (Figure S1, Tables S3 and S5).

**FIGURE 3:**
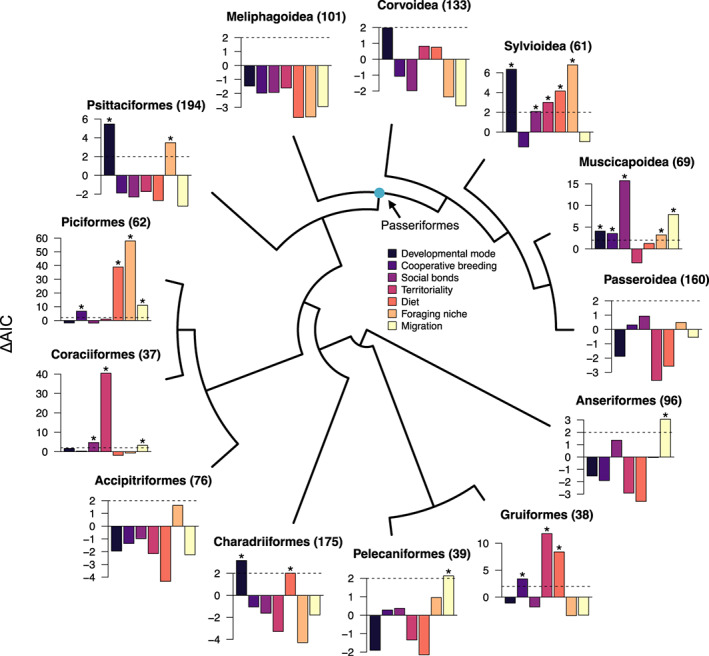
The relative importance of ecological and social factors for predicting relative brain size variation within major avian clades. Bar charts for each clade (*n* = 13) show the change in model support (ΔAIC value) when the given predictor was dropped from the model. Positive ΔAIC values indicate greater statistical support for the importance of a predictor, with values of ΔAIC > 2 (indicated by dashed lines) considered statistically significant (marked with an asterisk). The names of each clade (and number of sampled species) are shown in each case and the central tree schematic indicates the phylogenetic relationships among the groups (branch lengths not to scale). For details regarding factor levels for each variable and associated parameter estimates, see Table S6.

Repeating our analyses at lower taxonomic levels (i.e. within major avian clades) revealed substantial heterogeneity in the relative importance of social, ecological and developmental factors in driving brain size evolution in subsections of the avian phylogeny (Figure 3, Table S6). For example, despite relatively weak and/or non‐existence effects across all birds, we observed significant effects of cooperative breeding, dietary niche differences and migration on relative brain size differences in several avian clades (Gruiformes, Piciformes, Charadriiformes, Pelecaniformes, Sylvioidea, Muscicapoidea). These effects are indicated by large positive ΔAIC values when these predictors are dropped from clade‐specific models (Figure 3; factor levels and parameter estimates are shown in Table S6). In contrast, several clades showed significant impacts of variables also inferred to be important across all birds, including social bonding (Passeroidea, Coraciiformes, Muscicapoidea), territoriality (Gruiformes, Coraciiformes, Sylvioidea) and foraging niche (Piciformes, Psittaciformes, Muscicapoidea), yet often with idiosyncratic effects (Table S6). Finally, in three clades (Anseriformes, Accipitriformes, Meliphagoidea) we did not detect an effect of any factor in explaining relative brain size variation among their constituent species (Figure 3, Table S6). Again, results were similar using alternative ecological categorizations and/or including body mass as a covariate (Figure S2, Tables S7 and S9).

## 4 DISCUSSION

In this study, we have shown that ecological and social factors, as well as developmental constraints, play an important role in explaining variation in relative brain size across bird species. Our analyses demonstrate that across all birds there is a general relationship between relative brain size and variation in foraging niche, social bond strength, territoriality and developmental mode. However, despite these overarching effects, our analyses focusing on smaller avian clades reveal that there is considerable heterogeneity in the relative importance of such factors within different parts of the avian phylogeny, implying that the importance of ecological and social selection pressures for driving avian brain size evolution is often highly clade‐ and context‐specific.

Across all birds, we found that foraging niche was significantly correlated with brain size and explained more variation in brain size than all other social/ecological variables. Interestingly, however, we found no significant effect of dietary niche differences on brain size. Overington et al. (2009) similarly found that innovations in foraging mode were more frequent in birds with relatively larger brains, but food‐type innovations were not. They suggest this may reflect differences in opportunity, as species that eat diverse foods may be exposed to more novel food‐type opportunities, and thus dietary generalism need not select for increased brain size. Foraging niche, on the other hand, may select for increased brain size if exploiting new foraging niches requires complex manipulation (Heldstab et al., 2016). Day et al. (2005) found bower complexity was positively correlated with cerebellum size in bowerbirds. The cerebellum controls motor skills in birds (Sultan & Glickstein, 2007), so the relationship between foraging niche and total relative brain size we identify may reflect selection for complex motor skills primarily impacting the cerebellum and no other brain regions. However, the cerebellum typically accounts for less than 18% of total avian brain size (Iwaniuk & Hurd, 2005) and the majority of interspecific variation in relative brain size of birds and other amniotes is due to variation in the cerebral hemispheres (telencephalon) (Kverkova et al., 2022). Indeed, complex manipulation of food items requires sensorimotor integration, which would involve telencephalic brain regions (Shanahan et al., 2013). This highlights the need for further study into mosaic effects of brain size evolution as more data becomes available, particularly because different selection pressures may act on different brain regions without affecting overall brain size (Healy & Rowe, 2007), masking such effects in studies of total brain volume such as ours.

At face value, the lack of a relationship between diet and brain size across birds does not support the expensive brain hypothesis, as there is no evidence that species counter the costs of expensive brains via changes in food type. However, species with relatively large brains may meet the energy requirements of large brains through changes in caloric/nutrient intake within dietary categories, which is difficult to test at broad scales using currently available data. We also find no support for a relationship between brain size and migration, arguing against the idea that migratory species have reduced relative brain size due to a general trade‐off in energy allocation between brain tissue and locomotion (Isler & van Schaik, 2009). Interestingly, the results of a recent empirical study suggest that energetic trade‐offs between the brain and locomotion in birds can be largely overcome by switching to a less energetically costly flight mode (i.e. soaring rather than flapping) (Shiomi, 2022). This potentially helps to explain why, when looking across all birds (i.e. soarers and flappers) we find no evidence of link between migratory behaviour and brain size.

In terms of social variables, we find that across all birds social bond is a statistically important predictor of brain size in our models, but the direction of this relationship is non‐linear. Species considered to be highly social in our analysis do not have relatively larger brains than solitary species, and thus our results do not support the social brain hypothesis in birds. This result mirrors the findings of Fedorova et al. (2017), where living in stable social groups was associated with reduced brain size in woodpeckers. The authors argue that group‐living does not select for cognitive complexity in this group and suggest that sociality only selects for increased brain size in taxa where there is considerable inter‐individual competition for resources among (unrelated) individuals (e.g. primate societies [Byrne, 1996; Seyfarth & Cheney, 2002]). In contrast, relationships within group‐living bird species may be intrinsically cooperative, because such groups are typically comprised of closely related individuals and depend on cooperation among group members (Downing et al., 2020; Fedorova et al., 2017). This distinction between the importance of competitive versus cooperative interactions for determining brain size is bolstered by another of our findings that across all birds strongly territorial species have significantly larger brains than non‐territorial species. Territoriality in particular may be generally associated with increased brain size because of the potential fitness advantages of an increased auditory or spatial memory capacity (Araya‐Salas et al., 2018; Ikebuchi & Okanoya, 2000; Potts & Lewis, 2016). For example, male nightingales (*Luscinia megarhynchos*) alter their territorial defence behaviours in response to previous experiences with an intruder (Schmidt et al., 2007), providing further support for the idea that selection for memory in species engaging in frequent competitive interactions could be an important driver of avian brain size evolution.

On the other hand, another possible explanation for the non‐linear relationship between brain size and social bond is that the measure of social bond included in our model (strength of mate fidelity) does not effectively measure variation in social complexity in birds. Thus, the species categorized as highly social in our dataset may not actually experience heightened selection pressures for increased brain size due to social complexity. It is also possible that variation in social bond is not directly correlated with brain size and that the observed pattern emerges because social bond is correlated with another variable, such as parental care or mating system. Indeed, there is evidence that pair‐bondedness and monogamy are both important drivers of avian brain size (Shultz & Dunbar, 2010; West, 2014). More generally, Tobias et al. (2012) argue that sexual selection should be incorporated into the wider framework of social selection because it is driven by social interactions. Future research on avian brain size evolution may, therefore, benefit from including measures of sexual selection (e.g. mating system/parental care [García‐Peña et al., 2013]), as the nature of social interactions may be more important than the number or duration of social interactions in driving avian brain size evolution.

In addition to ecological and social factors, our analyses reaffirm the seemingly important role that developmental mode has in shaping relative brain size across birds. Specifically, using recently published, quantitative estimates of avian developmental mode variation (Ducatez & Field, 2021), we find a clear link between the degree of offspring precociality and smaller relative brain sizes across birds. Previous analyses have documented similar patterns using relatively coarse information on avian development (e.g. classifying species as altricial, semi‐precocial, precocial, etc.) (Bennett & Harvey, 1985; García‐Peña et al., 2013; Iwaniuk & Nelson, 2003; Shultz & Dunbar, 2010) and encouragingly our results demonstrate that these patterns hold using improved data. Together, these observations support the view that offspring precociality and the associated changes in offspring and adult life history (e.g. shorter duration of offspring embryonic development and reduced parental care) places severe constraints on the evolution of large brains in precocial species (Bennett & Harvey, 1985; García‐Peña et al., 2013; Iwaniuk & Nelson, 2003; Shultz & Dunbar, 2010). We also note that by controlling for these effects in our study, none of the associations between brain size, ecology and sociality we identify can be explained simply as the correlated effect of variation in offspring developmental mode across bird species.

An important additional feature of our results is that, despite these overarching trends, there appears to be considerable variation in the relative importance of such factors for explaining patterns of brain size evolution within distinct avian clades. For instance, across all species we identify a dominant role for particular variables (foraging ecology, social bonding and territoriality) in explaining patterns of brain size evolution. But at finer taxonomic scales (i.e. within orders), relationships with these factors appear far more heterogeneous, with some clades exhibiting relationships that mirror the overarching trends but many others showing more idiosyncratic relationships with other variables or no detectable relationships at all. There are several ways to interpret these findings. One interpretation is that, because the patterns we detect at the whole‐clade level are relatively weak (as indicated by relatively small partial *R* ^2^ values), the lack of similar effects in many clades simply reflects a general lack of power to detect such effects in smaller clades. While this is plausible, it could also be that avian clades generally lack the breadth of social and ecological trait variation responsible for driving relationships with brain size variation across all species. For example, it is well known that most ecological and life history variation in birds is partitioned among rather than within higher taxonomic levels (e.g. orders) (Cooney et al., 2017, 2020; Owens & Bennet, 1995; Pigot et al., 2020). This implies that the overarching importance of variables such as foraging ecology, social bonding, territoriality and developmental mode for explaining brain size evolution in birds is primarily driven by the considerable variation in these variables among major avian lineages, with comparatively small within‐clade effects. Nonetheless, despite these considerations, in many cases we do observe strong within‐clade effects of certain factors. Interestingly, in some cases these effects involved variables that are not identified as being important predictors of brain size variation across all birds (e.g. cooperative breeding and diet in Gruiformes and Piciformes). This suggests that, in some cases at least, selection on brain size can be highly clade‐ or context‐specific, generating patterns of brain size evolution that are not mirrored in other groups or across the avian radiation more generally. Understanding why the importance of ecological and social factors influencing brain size appears to vary among clades is a potentially fruitful avenue for further research, involving (for example) detailed comparisons of how clade life history and/or behaviour moderates the importance of ecological and social selection pressures on brain size.

In summary, our results reveal important impacts of both ecology and sociality in shaping brain size evolution in birds. While our selection of variables is not exhaustive and other dimensions of ecological and social variation are likely to be important (e.g. habitat complexity, environmental variability, flocking propensity, social learning, etc.) (Sayol et al., 2016), here we find that differences in foraging niche have an important role in explaining variation in relative brain size across bird species, and propose that this correlation may be driven by selection for complex motor skills. We also document strong effects of several social variables on avian brain size evolution, specifically social bond type and degree of territoriality, yet the direction of these effects provide little support for the classic social brain hypothesis of brain size evolution. Importantly, our work also highlights several important areas for future research. In particular, more work is required to understand how variation in ecological and social factors interact to shape selection on brain size, and whether selection acts to produce mosaic rather than whole brain size effects on brain size evolution. Our work also reveals the variable and potentially context‐specific ways in which ecological and social selection pressures may shape bird brain size evolution in avian clades, and highlights the need for more nuanced and focused comparative analyses in such groups using metrics that adequately capture the variation in selection pressures experienced by different avian species.

## AUTHOR CONTRIBUTIONS

**Jasmine L. Hardie:** Conceptualization (equal); data curation (equal); formal analysis (equal); investigation (equal); methodology (equal); project administration (equal); visualization (equal); writing – original draft (equal); writing – review and editing (equal). **Christopher R. Cooney:** Conceptualization (equal); data curation (equal); formal analysis (equal); funding acquisition (lead); investigation (equal); methodology (equal); project administration (equal); supervision (lead); visualization (equal); writing – original draft (equal); writing – review and editing (equal).

## FUNDING INFORMATION

This work was supported by a Natural Environmental Research Council Independent Research Fellowship to C.R.C [grant number NE/T01105X/1].

## CONFLICT OF INTEREST

The authors have no conflict of interest to declare.

## Supporting information


Figures S1

Tables S1

## Data Availability

The full dataset analysed in this study is available at https://doi.org/10.15131/shef.data.20937985.v2.
