# Supplementary material for: Sociality, ecology and developmental constraints predict variation in brain size across birds
Source: J Evol Biol. 2022 Nov 10;36(1):144–55. doi: 10.1111/jeb.14117 (PMC10100238; doi:10.1111/jeb.14117)
Supplement: Supplementary file 1 — Figures S1 [file JEB-36-144-s001.docx]

**Supporting Information for:**

**Sociality, ecology and developmental constraints predict variation in brain size across birds**

Jasmine L. Hardie* and Christopher R. Cooney*

* Joint corresponding authors: j.hardie@sheffield.ac.uk, c.cooney@sheffield.ac.uk

**Supplementary figures:**

**Figure S1. Plots showing the relationship of relative brain volume with developmental, social and ecological variables across bird species using finer ecological categorisations.** In all cases, box and whisker plots show the median (centre line) and interquartile range (box) of the data, the range of data which is within 1.5 times the interquartile range of the box (whiskers), and the position of outliers (points) that lie beyond this range. Values under each box give the number of species in each category. In F, letter codes represent ‘aquatic animals’ (Aa), ‘aquatic plants’ (Ap), ‘carrion’ (Ca), ‘fruit’ (Fr), ‘generalist’ (Ge), ‘invertebrates’ (In), ‘nectar’ (Ne), ‘seeds’ (Se), ‘terrestrial plants’ (Tp), ‘vertebrates’ (Ve). In G, letter codes represent ‘aerial sallying’ (Asa), ‘aerial screening’ (Asc), ’aquatic dive’ (Adi), ‘aquatic plunge’ (Apl), ‘aquatic surface’ (Asu), ‘arboreal gleaning’ (Agl), ‘bark gleaning’ (Bgl), ‘foraging generalist’ (Fgn), ‘ground foraging’ (Gfo).

**Figure S2. The relative importance of ecological, social and developmental factors for predicting relative brain size variation within major avian clades using finer ecological categorisations.** Bar charts for each clade (*n* = 13) show the change in model support (ΔAIC value) when the given predictor was dropped from the model. Positive ΔAIC values indicate greater statistical support for the importance of a predictor, with values of ΔAIC > 2 (indicated by dashed lines) considered statistically significant (marked with an asterisk). The names of each clade (and number of sampled species) are shown in each case and the central tree schematic indicates the phylogenetic relationships among the groups (branch lengths not to scale). For details regarding factor levels for each variable and associated parameter estimates, see Table S8.
